# Predictive Value of 48-hour Anion Gap Fluctuations in ICU Patients with Acute Kidney Injury: An Analysis based on MIMIC Database

**DOI:** 10.2174/0113816128385046250912044104

**Published:** 2025-10-07

**Authors:** Liling Hu, Shiva Khoshravesh, Qingquan Liu

**Affiliations:** 1 Department of Nephrology, the Second Hospital of Zhuzhou City, Zhuzhou, China;; 2 Department of the Second Clinical College, Tongji Hospital, Tongji Medical College, Huazhong University of Science and Technology, Wuhan, China;; 3 Department of Nephrology, Tongji Hospital, Tongji Medical College, Huazhong University of Science and Technology, Wuhan, China

**Keywords:** Anion gap, acute kidney injury, mortality, MIMIC database, intensive care unit, hypertension

## Abstract

**Introduction:**

Increasing anion gap (AG) correlates with both short- and long-term mortality in intensive care unit (ICU) patients with acute kidney injury (AKI). However, the relationship between AG fluctuations and AKI prognosis has been understudied. This study aims to evaluate the predictive value of AG fluctuations within the first 48 hours after ICU admission for renal recovery and 30-day all-cause mortality in AKI patients.

**Methods:**

Data were extracted from the Medical Information Mart for Intensive Care (MIMIC-IV, v2.2) database, including AKI patients aged 18 and older. A multifactorial Cox regression model was employed to assess the impact of AG fluctuations within 48 hours of ICU admission on mortality, adjusted using five models. Kaplan-Meier survival curves and curve-fitting analysis were used to illustrate the relationship between AG fluctuations and mortality risk.

**Results:**

A total of 15,438 patients with AKI were included, 57.0% of whom were male. The 30-day all-cause mortality rate was 19.19%. Patients were categorized into three groups based on AG fluctuations within the first 48 hours: <3 mmol/L, 3-5 mmol/L, and >5 mmol/L. Cox regression and survival analysis indicated a significantly higher 30-day mortality rate in the >5 mmol/L group (HR = 1.63; 95% CI = 1.50-1.77, *P*< 0.001), with the worst prognosis. Restricted cubic spline analysis revealed a nonlinear relationship between AG fluctuations and 30-day mortality risk.

**Discussion:**

The findings suggest that AG fluctuations during the first 48 hours of ICU admission are closely associated with adverse outcomes in AKI patients. Monitoring AG dynamics may aid clinicians in identifying high-risk patients and enhancing patient management by allowing for timely interventions that may improve prognosis.

**Conclusion:**

AG fluctuations within the first 48 hours of ICU admission are a key predictor of renal recovery and 30-day mortality in AKI patients. AG fluctuations greater than 5 mmol/L are significantly associated with increased mortality risk.

## 1 INTRODUCTION

Acute kidney injury (AKI) is a prevalent and serious complication in clinical practice, characterized by high incidence and mortality rates. Its impact is particularly significant in the intensive care unit (ICU), where severe AKI is associated with a notably elevated mortality rate [1, 2]. Epidemiological data suggest that AKI affects 10% to 20% of hospitalized patients, but this incidence can rise dramatically in ICU settings, reaching up to 50% or more [3, 4]. AKI is linked to a higher demand for renal replacement therapy, longer hospital stays, increased healthcare costs, and greater mortality both during hospitalization and after discharge [5, 6]. A meta-analysis has reported that the overall mortality rate in AKI patients is 23% [7], while large-scale prognostic studies reveal that AKI patients experience twice the annual mortality rate of those without AKI after discharge [8]. The etiology of AKI is highly heterogeneous, with significant variability in its incidence, underlying causes, and pathophysiology, particularly in ICU patients who often present with multiple comorbidities [9, 10]. Various prognostic markers have been proposed to predict AKI outcomes, including serum potassium, creatinine, cystatin C, and KIM-1, along with the RIFLE classification system for AKI [11-14]. Despite these efforts, no consensus has emerged regarding a definitive prognostic model.

The anion gap (AG), a calculated parameter widely used in diagnosing metabolic acidosis, has emerged as a potentially valuable tool for predicting AKI outcomes. Elevated AG levels have been linked to poor prognoses in various conditions such as heart disease, sepsis, and liver failure [15-18]. In AKI patients, particularly those in the ICU, research has demonstrated that elevated AG is a strong predictor of all-cause mortality [19, 20]. Studies involving real-world cohorts have shown that increased AG at ICU admission is associated with an increased risk of all-cause mortality of patients with acute myocardial infarction complicated by heart failure or pulmonary hypertension patients with sepsis [19, 21, 22]. However, the relationship between AG fluctuations and AKI prognosis has been understudied. Identifying a reliable, easy-to-measure biomarker that can inform prognosis and guide clinical decision-making would represent a major step forward in the management of AKI.

The Medical Information Mart for Intensive Care (MIMIC) database, a comprehensive repository of ICU patient data, provides an invaluable resource for investigating critical care outcomes, treatment strategies, and prognostic factors [23, 24]. This study aims to explore the clinical relevance of AG fluctuations during the first 48 hours of ICU admission and their association with renal recovery and 30-day mortality in AKI patients.

## 2 METHODS

### 2.1 Data Source

The data in this study were all obtained from the MIMIC-IV v2.2 database, which is the result of a collaboration between Beth Israel Deaconess Medical Center (BIDMC, Boston, Massachusetts, USA) and the Massachusetts Institute of Technology. The database contains 431,231 admission records and 73,181 ICU admission records from 2008 to 2019. This comprehensive database was compiled by a collaborative consortium that includes the Laboratory of Computational Physiology at the Massachusetts Institute of Technology, Beth Israel Deaconess Medical Center, Harvard Medical School, and Philips Healthcare [25]. Supported by substantial funding from the National Institutes of Health, the project underwent rigorous ethical review and approval from relevant Institutional Review Boards. The database represents a concerted effort to advance critical care research through the aggregation and detailed analysis of extensive clinical data. Personnel involved in data extraction were required to undergo specialized training and obtain certification (ID: 51474658) for database access and data retrieval. PostgreSQL software was utilized to interact with the MIMIC-IV database, and all data were extracted using structured query language (SQL) code [26]. The research was conducted in strict accordance with the ethical principles outlined in the Declaration of Helsinki.

### 2.2 Patient Selection

The study cohort was composed of adult patients (aged 18 years and older) diagnosed with AKI according to the Kidney Disease: Improving Global Outcomes (KDIGO) guidelines [27]. All patients included in the study were admitted to the ICU for more than 48 hours. Exclusion criteria included patients with multiple ICU admissions, those who died within 48 hours of admission, and those with incomplete essential data. This stringent selection process ensured the integrity of the dataset, and the patient recruitment process is outlined in Fig. (**1**).

### 2.3 Data Extraction

AG fluctuation was defined as the difference between the highest and lowest anion gap (AG) values within the first 48 hours of ICU admission. Patients were categorized into three groups based on tertiles of AG fluctuation during this time period: Low AG fluctuation: < 3 mmol/L, Moderate AG fluctuation: 3-5 mmol/L, High AG fluctuation: > 5 mmol/L [28]. AG were calculated using the formulae: AG (mmol/l) = (sodium + potassium) - (chloride + bicarbonate) [29, 30]. The albumin-corrected anion gap (ACAG) was determined using the formula: ACAG (mmol/l)=[4.4-albumin (g/dl)]×2.5+AG [29, 31]. Kidney function recovery was assessed by analyzing discharge urine output (UO) and serum creatinine (SCr) values from the MIMIC-IV database. Recovery of renal function was defined as SCr at discharge being ≤ 1.5 times the baseline value and UO greater than 0.5 ml/kg/h within 24 hours before discharge [32, 33].

### 2.4 Outcomes

The primary outcomes of the study were renal function recovery and 30-day all-cause mortality. Five statistical models were applied to assess these outcomes: Model 1: Unadjusted (no control for confounders). Model 2: Adjusted for demographic factors such as age, sex, and race. Model 3: Further adjusted for clinical factors including systolic blood pressure, diastolic blood pressure, hemoglobin, creatinine, and platelet levels. Model 4: Additional adjustments for comorbidities: SOFA score, myocardial infarction, congestive heart failure, diabetes, liver disease, chronic kidney disease, malignant tumors, chronic obstructive pulmonary disease (COPD), and sepsis. Model 5: Built upon Model 4 by adjusting for bicarbonate, blood glucose, blood calcium, blood potassium, blood sodium, and blood chloride levels.

### 2.5 Statistical Methods

Statistical analysis was conducted using R version 4.3.3. Continuous variables with normal distribution were presented as mean ± standard deviation (SD), while non-normally distributed variables were expressed as median (interquartile range). Categorical data were reported as percentages. Since less than 1% of the data were missing, mean imputation was employed to handle missing values. Multivariate logistic regression and Cox regression analyses were used to evaluate the association between AG fluctuations within the first 48 hours of ICU admission and the primary outcomes (renal function recovery and 30-day all-cause mortality). Kaplan-Meier survival curves were generated to analyze the impact of AG fluctuations on 30-day mortality. The correlation between the AG fluctuations in AKI patients and the risk of 30-day all-cause mortality was further explored using restricted cubic spline (RCS) analysis [34, 35]. A *p*-value of less than 0.05 was considered statistically significant.

## 3 RESULTS

### 3.1 Comparison of Clinical Baseline Characteristics

A total of 15,438 adult patients with AKI in the ICU were enrolled in the study. The median AG fluctuation was 4 mmol/L (range 2-6 mmol/L). The average age of the patients was 67.8 ± 15.6 years, with males comprising 57.0% (8,797 patients). AKI stage 3 had the highest prevalence, accounting for 33% of the cohort. Platelet counts showed a progressive decline across the three groups, while SOFA scores increased correspondingly. The proportion of patients with myocardial infarction and liver disease was higher in the group with the greatest AG fluctuations. No significant differences were observed in hospital stay duration or renal function recovery among the groups. However, the 30-day mortality rate varied significantly between the groups (*p <* 0.001), as shown in Table **1**.

### 3.2 Relationship Between AG Fluctuation and Renal Function Recovery and 30-Day Mortality in AKI Patients

In the univariate analysis, AG fluctuation greater than 5 mmol/L was found to be a risk factor for renal function recovery (HR = 0.73, *p <* 0.05) and 30-day all-cause mortality (HR = 1.63, *p <* 0.05). Multivariate regression analysis, adjusting for confounding factors such as age, sex, race, and comorbidities, confirmed the significance of AG fluctuations with respect to renal function recovery (HR = 0.80, *p <* 0.05) and 30-day mortality (HR = 1.47, *p <* 0.05). However, after further adjustment for ionic concentrations in the blood (Model 5), AG fluctuations were no longer associated with these outcomes, as presented in Table **2**. Furthermore, we also evaluated whether ACAG fluctuations within 48 hours were independent risk factors for renal function recovery and 30-day mortality. Due to the absence of albumin values in 7,636 AKI patients, AVAG analysis was only conducted in 7,802 AKI patients. After conducting univariate and multivariate Cox analyses, we found that the results obtained by ACAG as an independent factor were consistent with those obtained by AG as an independent factor (Table **S1**), This indicated that the results obtained by taking AG as the subject of the study were robust.

### 3.3 Survival Analysis of AKI Patients Within 30 Days Based on AG Fluctuations

Kaplan-Meier survival curves were constructed according to the tertiles of AG fluctuations. The results indicated that patients with AG fluctuations >5 mmol/L had a significantly lower cumulative 30-day survival rate compared to the other two groups, with a highly significant difference (*p <* 0.0001) (Fig. **2**). Moreover, we further analyzed the impact of AG fluctuations on the survival period of AKI patients based on AKI stage or sepsis status. The results indicated that regardless of AKI stage 1, AKI stage 2, or AKI stage 3, the cumulative 30-day survival rate of AKI patients with AG fluctuation >5 mmol/L was significantly lower than that of the other two groups. Similarly, whether it was AKI caused by sepsis or AKI not caused by sepsis, it was found that the survival rate of AKI patients with AG fluctuation >5 mmol/L was decreased significantly (Fig. **S1**).

### 3.4 Restricted Cubic Splines Analysis of 30-Day Mortality in AKI Patients Based on AG Fluctuations

To better capture the nonlinear relationship between AG fluctuations and patient outcomes, RCS analysis was performed. Nodes were defined within the data range, and polynomial functions were applied to fit the data between these nodes. The analysis revealed a nonlinear association between AG fluctuation values and the 30-day mortality rate in AKI patients (P non-linear = 0.008), as illustrated in Fig. (**3**). Furthermore, we found that this nonlinear association this nonlinear relationship is not affected by the stage of AKI and whether AKI was caused by sepsis (Fig. **S2**).

## 4 DISCUSSION

This study aimed to determine whether early variations in the AG could serve as an indicator of AKI outcomes in ICU patients, thereby advancing our understanding of AKI management and decision making in the identification of potential avenues for early intervention in critical care. In this study, we found that AG fluctuations within the first 48 hours of ICU admission are a key predictor of renal recovery and 30-day mortality in AKI patients by collecting data from the MIMIC-IV database. AG fluctuations greater than 5 mmol/L are significantly associated with increased mortality risk by Cox regression and survival analysis. While these findings align with prior studies linking elevated AG to poor outcomes in critically ill populations [36, 37].

AG is a key indicator for evaluating acid-base balance, which maintains its function within the standard range under normal circumstances, providing valuable insights into acid-base status [38, 39]. AG was observed to be elevated in the vast majority of cases in patients with acute kidney injury [40, 41]. In AKI, impaired renal acid excretion and reduced bicarbonate regeneration compromise the body’s capacity to neutralize accumulating anions (*e.g*., lactate, phosphate) [42, 43]. Elevated ACAG levels (> 20 mmol/L) at ICU admission were an independent risk indicator associated with 30-day and 360-day all-cause mortality in critically ill patients with AKI [41, 44]. Clinical practice research has found that AG level can be used as a valuable predictive indicator for the risk of death during hospitalization in patients with SA-AKI [45, 46]. Static AG values capture a single time point of metabolic status, whereas AG fluctuations reflect the inability of physiological buffering systems to maintain equilibrium under stress. Frequent AG oscillations may signal exhaustion of buffering reserves, as the kidneys and respiratory system struggle to compensate for ongoing metabolic insults [31, 47, 48]. Lactate is a major contributor to AG elevation, and transient AG peaks may represent intermittent tissue hypoxia [49-52]. The transient change of AG value cannot fully represent the severity of the disease or progression of the disease. Studies in septic shock have shown that lactate variability—rather than absolute values—predicts mortality [53, 54]. Although existing studies have reported that the levels of AG or ACAG are associated with the risk of AKI in patients. However, few studies have explored the correlation between the dynamic changes of AG during hospitalization in critically ill patients and the prognosis of AKI. The dynamic changes of AG can serve as markers of insufficient tissue perfusion and metabolic imbalance [48, 55]. AG fluctuations reflect the dynamic process of metabolic disorders and can better reflect the dynamic changes of compensatory capacity than a single measurement value [56, 57]. Renal acid excretion disorders and insufficient respiratory compensation jointly lead to sharp fluctuations in AG. Monitoring the dynamic changes of AG can indicate the changing trend of multiple organ dysfunction [58-60]. Therefore, dynamic AG monitoring has greater prognostic value than static values. Our findings revealed a clear association between higher AG fluctuations and adverse outcomes. Specifically, patients with AG fluctuations greater than 5 mmol/L had a lower likelihood of renal recovery and a significantly higher 30-day mortality rate. The initial univariate analysis indicated that AG fluctuations above this threshold were predictive of both poor renal outcomes and increased mortality risk. Multivariate Cox regression analysis, which adjusted for non-ionic confounders such as age, sex, and comorbidities, maintained the significance of AG fluctuations in relation to both outcomes. However, after further adjusting for serum ionic concentrations, AG fluctuations no longer predicted outcomes. The loss of AG’s predictive value in Model 5 (after adjusting for ions) underscores that its prognostic utility is mediated through these downstream electrolyte disturbances. This discovery reveals that the prognostic value of AG fluctuations does not exist independently but is indirectly mediated through metabolic disorders and the severity of systemic diseases. The observational clinical study indicated that AG fluctuations often parallel shifts in serum electrolytes. Fluctuations in AG may thus represent dynamic shifts in these cryptic anions. Although the direct effect of AG fluctuations in Model 5 disappeared, its dynamic changes still have clinical warning significance. Targeting the specific abnormalities behind AG fluctuations (such as correcting acidosis and managing hyperkalemia) may be more effective than focusing on AG itself [61, 62]. Based on the properties of AG, it also highlights the importance of AG as an indicator of electrolyte disturbances in AKI patients. This discovery calls on clinicians to combine electrolyte profiles and organ function assessment when interpreting AG, rather than relying on AG values in isolation.

The ICU setting presents a complex environment, where patients frequently experience multiple organ dysfunction, systemic inflammatory response syndrome, and hemodynamic instability [63]. These factors contribute to inadequate renal perfusion and the use of nephrotoxic drugs, further impairing kidney function and perpetuating a cycle of renal decline [64]. Reduced kidney function not only leads to electrolyte imbalances but also precipitates metabolic acidosis, as the kidneys are unable to efficiently clear toxins and acidic metabolites. This creates a harmful feedback loop, where accumulating toxins further damages the kidneys [48, 65]. Even mild reductions in glomerular filtration rate can result in the retention of non-chloride anions, a phenomenon that contributes to elevated AG values in ICU patients [31, 66]. In AKI patients, the accumulation of anions such as lactate and phosphate, combined with sodium and water retention, significantly alters the AG, reflecting the severity of electrolyte and metabolic disturbances. Moreover, ICU patients frequently experience elevated physiological stress, which triggers sympathetic nervous system activation, accelerating glycolysis and disrupting bioenergetics, leading to elevated lactate levels [67]. Research shows that lactate and ketone bodies contribute to over 60% of AG increases [68], reinforcing the role of AG as a marker of metabolic imbalance. Fluctuations in AG, therefore, offer valuable insights into the metabolic and electrolyte status of critically ill patients, providing clinicians with a practical tool to monitor disease progression and the efficacy of therapeutic interventions.

In recent years, AG has gained recognition as a useful serological marker due to its ease of calculation and its predictive power in assessing mortality risk [69]. Elevated AG levels have been consistently linked with poor outcomes, particularly in AKI patients in the ICU [15, 16]. Previous studies have identified an AG threshold of 14 mmol/L or higher as a significant predictor of 28-day mortality in ICU patients with AKI [45]. Real-world studies have further demonstrated the relationship between elevated AG levels are associated with the 30-day, 90-day, and 365-day mortality rates of AKI patients in the ICU [19]. While much of the existing literature has focused on static AG values, our study highlights the prognostic importance of monitoring AG fluctuations over short periods. Specifically, our results indicate that AG fluctuations greater than 5 mmol/L are associated with significantly increased mortality risk, underscoring the need for clinicians to track not only absolute AG levels but also their dynamic changes in ICU patients. Patients with AKI are at high risk for metabolic acidosis due to impaired acid excretion, increased lactic acid production from infections or hypoxia, and conditions such as diabetic ketoacidosis [70-72]. Metabolic acidosis, particularly the high AG variant, is associated with worse clinical outcomes, including higher mortality rates and longer ICU stays [73]. Not only does AKI contribute to the development of metabolic acidosis, but growing evidence suggests that acidosis itself can further worsen renal function [65, 74]. AG plays a critical role in distinguishing between different types of metabolic acidosis, allowing clinicians to narrow the differential diagnosis and detect concurrent disturbances in acid-base balance [75]. Our study reinforces the importance of AG in this context, emphasizing that fluctuations in AG provide additional prognostic value, guiding more tailored and responsive management strategies in critically ill AKI patients.

This study is strengthened by its large sample size and the use of the MIMIC-IV database, which offers a rich, real-world dataset. However, there are several limitations to consider. First, the retrospective nature of this study provides a lower level of evidence compared to prospective cohort studies or randomized controlled trials. Second, we did not make ACAG as main subject of this study. Serum albumin contributes significantly to the plasma’s buffering capacity by binding to cations. This binding propensity, in turn, impacts the calculated anion gap [36, 38]. However, our research found that whether AG was taken as the main research object or ACAG was taken as the main research object, the conclusions obtained from both were consistent, indicating that our research results were robust. Lastly, the retrospective design of the MIMIC-IV database introduces potential residual confounding, despite rigorous adjustment for demographic, clinical, and biochemical variables. Unmeasured confounders, such as fluid balance, acid-base therapies (*e.g*., sodium bicarbonate administration), and nutritional status, may influence AG values and their interpretation. For instance, aggressive fluid resuscitation or diuretic use could alter electrolyte concentrations, indirectly affecting AG calculations. Interventions targeting acid-base balance might transiently mask true metabolic disturbances, thereby biasing the observed associations between AG fluctuations and outcomes. Nutritional factors, including hypoalbuminemia or protein intake, could further confound AG measurements. Future prospective studies incorporating detailed data on fluid management, therapeutic interventions, and nutritional parameters are warranted to validate our findings.

## CONCLUSION

AG fluctuations during the first 48 hours of ICU admission are closely associated with adverse outcomes in AKI patients, primarily reflecting underlying metabolic disturbances and systemic disease severity. While AG variability ≥5.00 mmol/L predicts short-term mortality in unadjusted analyses, this association is mediated by electrolyte imbalances and multi-organ dysfunction. Monitoring AG dynamics may aid clinicians in identifying high-risk patients and enhancing patient management by allowing for timely interventions that may improve prognosis.

## Figures and Tables

**Fig. (1):**
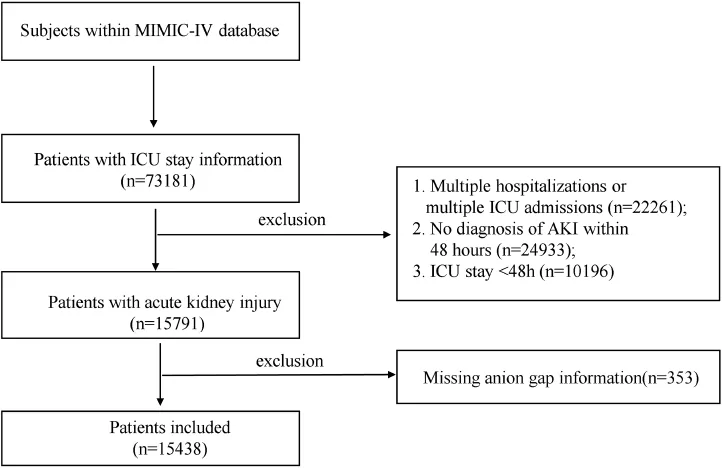
Flow chart for participant inclusion and exclusion. MIMIC III: Multiparameter Intelligent Monitoring in Intensive Care Database III; ICU: intensive care unit.

**Fig. (2):**
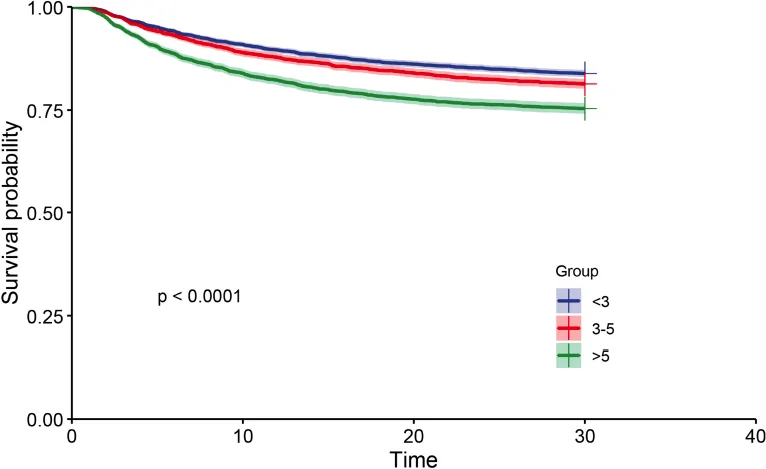
30-days Kaplan-Meier survival curve of AKI patients in subgroups with different anion gap fluctuations. **Abbreviations**: AKI: acute kidney injury, AG: anion gap.

**Fig. (3):**
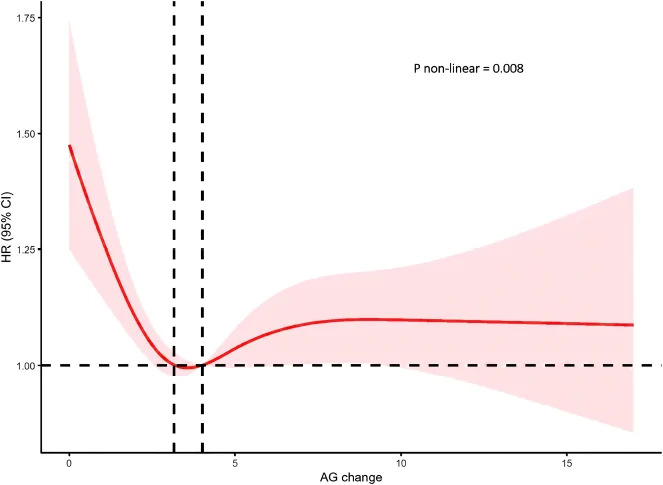
Association between fluctuating serum anion gap values and 30-days all-cause mortality risk in patients with AKI. **Abbreviations**: AKI: acute kidney injury; AG: anion gap; HR: hazard ratio.

**Table 1:** Population characteristics of AKI patients grouped by different AG fluctuation values.

**Variables**	**AG Fluctuation Values (mmol/L)**			***P*-value**
	**<3 (n=6904)**	**3-5 (n=4237)**	**>5 (n=4297)**	
**Gender, n (%)**	-	-	-	0.034
Female	3048 (44.1%)	1795 (42.4%)	1798 (41.8%)	-
Male	3856 (55.9%)	2442 (57.6%)	2499 (58.2%)	-
Age (years)	69.1 ± 15.0	68.1 ± 15.6	65.6 ± 16.3	<.001
Race, n (%)	-	-	-	<.001
Asians	142 (2.1%)	82 (1.9%)	129 (3%)	-
Black	541 (7.8%)	350 (8.3%)	390 (9.1%)	-
Hispanic	195 (2.8%)	123 (2.9%)	138 (3.2%)	-
White	4824 (69.9%)	2825 (66.7%)	2745 (63.9%)	-
Other Race	248 (3.6%)	169 (4%)	186 (4.3%)	-
Unknown	954 (13.8%)	688 (16.2%)	709 (16.5%)	-
**AKI Staging**	-	-	-	<.001
1	1833 (26.5%)	1093 (25.8%)	974 (22.7%)	-
2	3794 (55%)	2146 (50.6%)	1906 (44.4%)	-
3	1277 (18.5%)	998 (23.6%)	1417 (33%)	-
Systolic pressure, mmHg	117.6 ± 15.3	116.2 ± 15.1	114.8 ± 14.9	<.001
Diastolic pressure, mmHg	61.6 ± 11.8	61.9 ± 12.6	62.4 ± 12.2	0.008
SOFA score	5 (3~8)	6 (4~9)	8 (5~11)	<.001
**Laboratory Parameters**				
Creatinine, mg/dl	0.7(0.6~1)	0.8(0.6~1)	0.8(0.6~1.05)	<.001
Urea nitrogen, mg/dl	18 (13~28)	19(13~32)	21(14~35)	<.001
Hematocrit, %	30.4 ± 7.9	30.0 ± 7.1	29.8 ± 7.5	<.001
Hemoglobin, g/dL	9.8 (8.4~11.5)	9.7 (8.3~11.4)	9.7(8.1~11.5)	0.001
Platelet, ×10^9^/L	171(122~231)	164 (114~227)	152 (100~214)	<.001
Leukocyte, ×10^9^/L	9.7(7.2~12.83)	9.9 (7~13.3)	9.8 (7~13.3)	0.934
Bicarbonate, mmol/L	22.8 ± 6.7	21.4 ± 4.1	19.0 ± 4.6	<.001
Sodium ion, mmol/L	137.3 ± 4.2	136.7 ± 4.5	136.2 ± 5.2	<.001
Potassium, mmol/L	4.0 ± 0.5	3.9 ± 0.6	3.9 ± 0.6	<.001
Serum calcium, mmol/L	2.1 (2.0~2.2)	2.0 (1.9~2.2)	2.0 (1.8~2.1)	<.001
Chloride ion, mmol/L	102.9 ± 5.7	102.1 ± 5.8	101.2 ± 6.4	<.001
Glucose, mg/dL	119.6 ± 31.6	119.2 ± 32.8	119.6 ± 36.6	0.792
Fluctuation value of AG over 48 hours, mmol/L	2.0 ± 1.0	4.4 ± 0.5	8.2 ± 2.5	<.001
Urine volume, mL	1370(900~2010)	1345(835~2045)	1229(687~1995)	<.001
**Comorbidities**				
Myocardial infarction	1329 (19.2%)	845 (19.9%)	910 (21.2%)	0.046
Heart failure	2219 (32.1%)	1487 (35.1%)	1469 (34.2%)	0.003
COPD	1962 (28.4%)	1152 (27.2%)	1080 (25.1%)	<.001
CKD	1503 (21.8%)	991 (23.4%)	1035 (24.1%)	0.011
Malignancy	847 (12.3%)	587 (13.9%)	567 (13.2%)	0.046
Diabetes	2117 (30.7%)	1293 (30.5%)	1450 (33.7%)	<.001
Liver disease	688 (10%)	605 (14.3%)	854 (19.9%)	<.001
Sepsis	4312 (62.5%)	2856 (67.4%)	3248 (75.6%)	<.001
Total length of hospitalization, days	8.99 (6.1014.7)	10.19 (6.63~16.70)	10.82 (6.76~18.66)	<.001
Length of ICU hospitalization, days	3.63 (2.61~6)	4(2.78~7.11)	4.6(3~8.76)	<.001
Recovery of renal function, n (%)	3527 (51.1%)	2025 (47.8%)	1855 (43.2%)	<.001
30-day mortality rate	1114 (16.1%)	790 (18.6%)	1058 (24.6%)	<.001

**Abbreviations**: AKI: acute kidney injury, CKD: Chronic kidney disease, MIMIC-IV: Medical Information Mart for Intensive Care-IV, AG: anion gap, COPD: chronic obstructive pulmonary disease, ICU: intensive care unit, SOFA: sequential organ failure assessment.

**Table 2:** Regression analysis of AG fluctuation within 48 hours with renal function recovery and 30-day mortality rate.

**Characteristics**	**Model 1**		**Model 2**		**Model 3**		**Model 4**		**Model 5**	
	**HR (95% CI)**	***P***	**HR (95% CI)**	***P***	**HR (95% CI)**	***P***	**HR (95% CI)**	***P***	**HR (95% CI)**	***P***
**Renal Function Recovery**										
Fluctuation value of AG (mmol/L)	0.95 (0.94-0.96)	<0.001	0.95 (0.94-0.96)	<0.001	0.95 (0.94-0.96)	<0.001	0.97 (0.96-0.98)	<0.001	1.00 (0.99-1.01)	0.968
Fluctuation value of AG (mmol/L)	-	-	-	-	-	-	-	-	-	-
<3	(Ref)		(Ref)		(Ref)		(Ref)		(Ref)	-
3-5	0.88 (0.81-0.95)	<0.001	0.88 (0.81-0.95)	<0.001	0.89 (0.83-0.97)	0.004	0.93 (0.86-1.00)	0.09	1.01 (0.93-1.09)	0.825
>5	0.73 (0.67-0.78)	<0.001	0.73 (0.67-0.78)	<0.001	0.75 (0.70-0.81)	<0.001	0.82 (0.76-0.89)	<0.001	1.01 (0.93-1.10)	0.805
**30-day Mortality Rate**										
Fluctuation value of AG (mmol/L)	1.08 (1.06-1.09)	<0.001	1.09 (1.08-1.10)	<0.001	1.07 (1.06-1.09)	<0.001	1.05 (1.04-1.06)	<0.001	0.99 (0.98-1.01)	0.313
Fluctuation value of AG (mmol/L)	-	-	-	-	-	-	-	-	-	-
<3	(Ref)		(Ref)		(Ref)		(Ref)		(Ref)	
3-5	1.17 (1.07-1.28)	<0.001	1.18 (1.07-1.29)	<0.001	1.14 (1.04-1.25)	0.005	1.05 (0.96-1.16)	0.261	0.92 (0.84-1.01)	0.072
>5	1.63 (1.50-1.77)	<0.001	1.72 (1.58-1.88)	<0.001	1.58 (1.45-1.72)	<0.001	1.39 (1.27-1.51)	<0.001	0.96 (0.88-1.06)	0.454

**Note**: Model 1: No control of any confounding factors; Model 2: Controlling for confounding factors: age, gender, race, etc; Model 3: Confounding factors: systolic and diastolic blood pressure, creatinine, hemoglobin and platelets were controlled on the basis of model 2; Model 4: Confounders: SOFA score, myocardial infarction, heart failure, diabetes, liver disease, CKD, COPD, malignancy and sepsis were controlled for in Model III; Model 5: Bicarbonate, glucose, calcium, potassium, sodium, and chloride were adjusted from Model 4.

**Abbreviations**: Ref: reference, HR: hazard ratio, CI: confidence interval, AG: anion gap, CKD: chronic kidney disease, COPD: chronic obstructive pulmonary disease.

## Data Availability

The data and supportive information are available within the article.

## LIST OF ABBREVIATIONS

ACAG: Albumin-corrected Anion Gap
AG: Anion Gap
AKI: Acute Kidney Injury
ICU: Intensive Care Unit
KDIGO: Kidney Disease: Improving Global Outcomes
MIMIC: Medical Information Mart for Intensive Care
RCS: Restricted Cubic Spline
SCr: Serum Creatinine
SD: Standard Deviation
UO: Urine Output

## STANDARDS OF REPORTING

STROBE guidelines were followed.
