# Supplementary material for: Predictive Value of 48-hour Anion Gap Fluctuations in ICU Patients with Acute Kidney Injury: An Analysis based on MIMIC Database
Source: Curr Pharm Des. 2025 Oct 7;32(29):2450–9. doi: 10.2174/0113816128385046250912044104 (PMC13678608; doi:10.2174/0113816128385046250912044104)
Supplement: Supplementary file 1 [file CPD-32-29-2450_SD1.pdf]

Supplementary Material

Predictive Value of 48-hour Anion Gap Fluctuations in ICU Patients with Acute Kidney Injury: An Analysis based on MIMIC Database

Liling Hu[1], Shiva Khoshravesh[2] and Qingquan Liu[3,*]

[1]Department of Nephrology, the Second Hospital of Zhuzhou City, Zhuzhou, China; [2]Department of the Second Clinical College, Tongji Hospital, Tongji Medical College, Huazhong University of Science and Technology, Wuhan, China; [3]Department of Nephrology, Tongji Hospital, Tongji Medical College, Huazhong University of Science and Technology, Wuhan, China

Supplemental table 1 Regression analysis of ACAG fluctuation within 48 hours with renal function recovery and 30-day mortality rate.

Characteristics	Model 1		Model 2		Model 3		Model 4		Model 5	
	HR (95% CI)	P	HR (95% CI)	P	HR (95% CI)	P	HR (95% CI)	P	HR (95% CI)	P
**Renal function recovery**										
Fluctuation value of AG (mmol/L)	0.96 (0.95-0.97)	<0.001	0.96 (0.95-0.97)	<0.001	0.97 (0.95-0.98)	<0.001	0.98 (0.97-0.99)	0.008	1.01 (0.99-1.02)	0.497
Fluctuation value of AG (mmol/L)										
<3	(Ref)		(Ref)		(Ref)		(Ref)		(Ref)	
3-5	0.84 (0.76-0.94)	0.001	0.84 (0.76-0.93)	0.001	0.86 (0.78-0.96)	0.007	0.90 (0.81-1.01)	0.033	0.98 (0.88-1.10)	0.741
>5	0.75 (0.67-0.84)	<0.001	0.75 (0.66-0.84)	<0.001	0.79 (0.70-0.89)	<0.001	0.84 (0.74-0.94)	0.004	1.03 (0.91-1.18)	0.868
**30-day mortality rate**										
Fluctuation value of AG (mmol/L)	1.06 (1.04-1.07)	<0.001	1.07 (1.05-1.08)	<0.001	1.05 (1.04-1.06)	<0.001	1.04 (1.03-1.05)	<0.001	1.00 (0.98-1.01)	0.868
Fluctuation value of AG (mmol/L)										
<3	(Ref)		(Ref)		(Ref)		(Ref)		(Ref)	
3-5	1.12 (1.01-1.24)	0.038	1.13 (1.02-1.25)	0.024	1.08 (0.97-1.20)	0.152	1.03 (0.92-1.14)	0.633	0.90 (0.81-1.01)	0.065
>5	1.44 (1.29-1.61)	<0.001	1.55 (1.39-1.74)	<0.001	1.38 (1.23-1.54)	<0.001	1.26 (1.13-1.42)	<0.001	0.94 (0.83-1.07)	0.331

Model 1: No control of any confounding factors;
Model 2: Controlling for confounding factors: age, gender, race;
Model 3: Confounding factors: systolic and diastolic blood pressure, creatinine, hemoglobin and platelets were controlled on the basis of model 2;
Model 4: Confounders: SOFA score, myocardial infarction, heart failure, diabetes, liver disease, CKD, COPD, malignancy and sepsis were controlled for in Model III;
Model 5: Bicarbonate, glucose, calcium, potassium, sodium, and chloride were adjusted from Model 4.
ACAG: Ref: reference, HR: hazard ratio, CI: confidence interval, AG: anion gap, CKD: chronic kidney disease, COPD: chronic obstructive pulmonary disease.

1873-4286/26 $65.00+.00 **© 2026 Bentham Science Publishers**

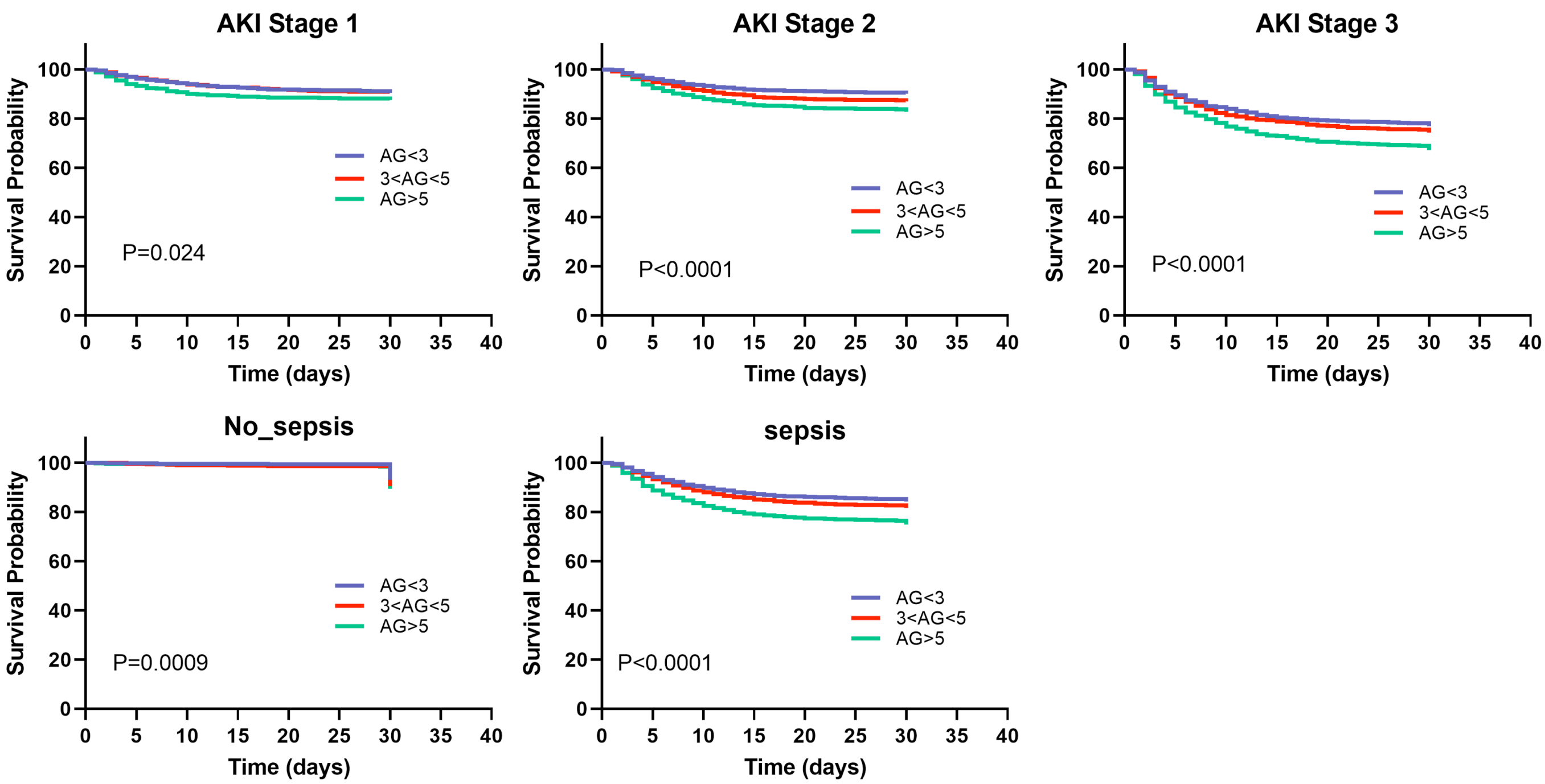

Fig. (S1). The 30-day Kaplan-Meier survival curves of AKI patients in subgroups with different anion gap fluctuations were analyzed hierarchically based on AKI staging and sepsis status. AKI: acute kidney injury.

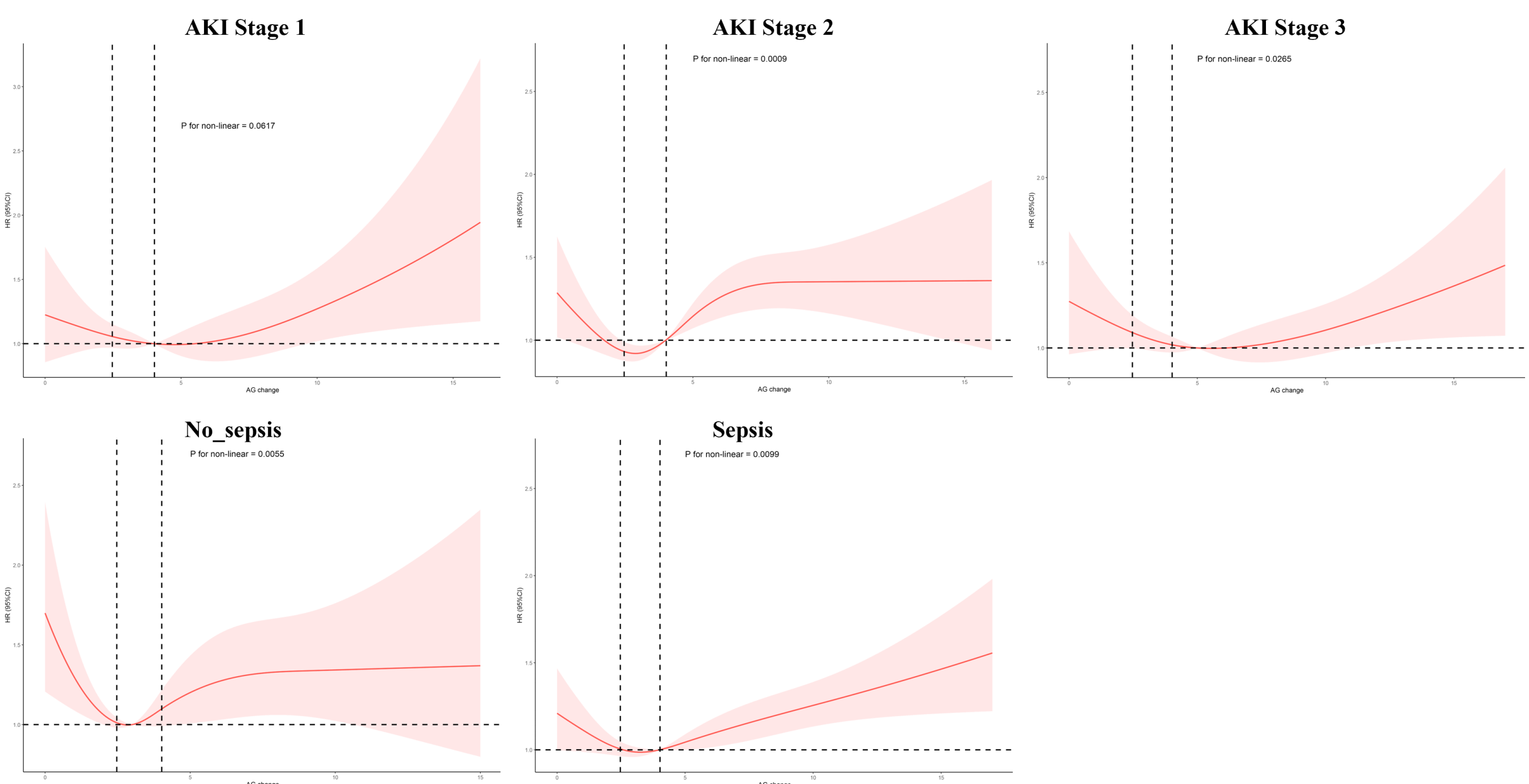

Fig. (S2). Association between fluctuating serum anion gap values and 30-days all-cause mortality risk in patients with AKI based on AKI staging and sepsis status. AKI: acute kidney injury; AG: anion gap; HR: hazard ratio.
